# Animal models for diabetic kidney disease: perspectives and prospects

**DOI:** 10.3389/fvets.2026.1805014

**Published:** 2026-05-04

**Authors:** Min Zeng, Qin Gong, Hua Shen, Long Chen, Wanxia Zhang

**Affiliations:** 1 The Eighth Clinical Medical College of Guangzhou University of Chinese Medicine, Foshan Hospital of Traditional Chinese Medicine, Foshan, China; 2 Guangdong Provincial Second Hospital of Traditional Chinese Medicine, Guangzhou, China; 3 The First Dongguan Affiliated Hospital of Guangdong Medical University, Dongguan, China

**Keywords:** animal models, diabetic kidney disease, streptozotocin, modeling methods, type 2 diabetes

## Abstract

Diabetic kidney disease (DKD) is one of the most severe complications of diabetes. Animal models of DKD play a crucial role in elucidating disease pathogenesis, exploring therapeutic strategies, and developing pharmacological interventions. This article systematically reviews the methodologies for establishing the four main categories of DKD animal models (induced, spontaneous, genetically engineered, and disease-syndrome combination models). We summarize the advantages, disadvantages, and inherent limitations of each model type. For each category, feasible optimization strategies are proposed. We advocate for the establishment of a forward-looking, “purpose-oriented” research paradigm. This paradigm aims to guide researchers in selecting models more precisely, optimizing them more proactively, and aligning evaluation criteria with clinical practice, thereby significantly enhancing the success rate and translational value of DKD research.

## 1 Introduction

Diabetic kidney disease (DKD) is one of the most severe microvascular complications of diabetes and a major cause of end-stage renal disease (ESRD) worldwide, imposing a substantial healthcare and economic burden on society (1, 2). The pathogenesis of DKD is extremely complex, resulting from the interplay of multiple factors, including metabolic disturbances and hemodynamic abnormalities under prolonged hyperglycemia, ultimately leading to progressive glomerulosclerosis and tubulointerstitial fibrosis (3–6). Due to the incomplete understanding of its mechanisms, the treatment of DKD remains a significant challenge. A large number of patients still progress to ESRD despite existing interventions (7–9). Animal models serve as a crucial platform for exploring disease mechanisms, identifying drug targets, and validating therapeutic strategies (10, 11). Developing animal models that can accurately simulate the key features of DKD is essential for elucidating its early pathogenesis, identifying diagnostic biomarkers, and developing effective intervention strategies. However, the translation of research findings from current DKD animal models to clinical applications continues to face major challenges (10–12).

This dilemma highlights the limitations of existing models in replicating the complex course of human DKD. Therefore, it is critical to evaluate these models and propose an optimization framework. This article aims to systematically analyze the advantages and limitations of various models, provide targeted optimization strategies, and guide the precise selection, proactive optimization, and clinically aligned evaluation of models, thereby enhancing the value of translational research.

## 2 Chemically and physically induced models

### 2.1 Streptozotocin (STZ)-induced type 1 diabetes (T1D)-like model

#### 2.1.1 Mechanisms

STZ enters pancreatic *β*-cells via glucose transporter 2 (GLUT2). Its metabolites induce DNA alkylation and strand breaks, leading to overactivation of poly (ADP-ribose) polymerase (PARP), subsequent NAD⁺ depletion, and energy metabolism impairment, ultimately causing *β*-cell apoptosis and absolute insulin deficiency with hyperglycemia. The sustained hyperglycemic state subsequently initiates and accelerates renal injury through metabolic disturbances, hemodynamic alterations, and oxidative stress. Of note, high-dose STZ also exerts direct cytotoxicity on renal tubular epithelial cells via DNA damage and oxidative stress mechanisms, which may introduce non-specific renal injury and confound the interpretation of DKD-specific pathological changes (13, 14).

#### 2.1.2 Modeling method

Through multiple low doses (mice: 40–55 mg/kg/day for 5 consecutive days; rats: 30–40 mg/kg/day for 3–5 consecutive days) or a single high dose (mice: 150–200 mg/kg; rats: 55–75 mg/kg) intraperitoneal injection of STZ, simulating autoimmune *β*-cell damage or direct toxic destruction, respectively, inducing absolute insulin deficiency and hyperglycemia (15–21).

#### 2.1.3 Advantages and disadvantages

This model is simple to operate, low cost, fast modeling, and has stable hyperglycemia, suitable for large-scale drug screening; however, its etiology is distorted (inconsistent with the autoimmune origin of human T1D), renal pathological phenotypes are superficial (mostly stagnating at early glomerular hypertrophy and microalbuminuria, rarely progressing to late-stage glomerulosclerosis, interstitial fibrosis, and progressive decline in glomerular filtration rate (GFR)), and high-dose STZ has direct toxicity to renal tubules.

#### 2.1.4 Optimization strategies

Prolong the observation period or use middle-aged animals to utilize the superimposed effect of aging; develop non-surgical acceleration methods such as continuous infusion of Angiotensin II (Ang II) to increase intraglomerular pressure.

### 2.2 High-fat diet combined with low-dose STZ-induced type 2 diabetes (T2D)-like model

#### 2.2.1 Mechanisms

Prolonged high-fat diet induces peripheral insulin resistance and obesity, placing pancreatic *β*-cells under compensatory hypersecretory stress. Subsequent low-dose STZ administration partially damages β-cells, leading to insufficient insulin secretion and overt hyperglycemia. This sequence recapitulates the pathophysiological process of human T2D, characterized by metabolic dysfunction preceding *β*-cell decompensation. Hyperglycemia and insulin resistance synergistically accelerate renal injury through metabolic disturbances, hemodynamic alterations, and inflammatory pathways (22, 23).

#### 2.2.2 Modeling method

Animals first receive a high-fat diet (HFD) for 4–8 weeks to induce insulin resistance (IR) and obesity, followed by injection of low-dose STZ (rats 30–35 mg/kg, mice 40–50 mg/kg) or multiple low-dose regimens (40–50 mg/kg/day for 5 consecutive days) to induce overt hyperglycemia and early kidney damage (22, 24–26).

#### 2.2.3 Advantages and disadvantages

Better simulates the metabolic characteristics of T2D (obesity, insulin resistance, *β*-cell dysfunction), closer to common clinical etiology; however, renal lesions are still mainly early manifestations (glomerular hypertrophy, mesangial matrix expansion), late fibrosis phenotypes are insufficient, and animals often die from acute metabolic complications.

#### 2.2.4 Optimization strategies

Finely adjust the combination of HFD feeding time and STZ dose according to research needs; superimpose a high-salt diet (HSD) to induce hypertension and construct a “metabolic syndrome (MetS)” composite model; change to a high-protein diet (HPD) after diabetes modeling to accelerate renal fibrosis progression.

### 2.3 STZ combined with nicotinamide (NAD)-induced T2D-like model

#### 2.3.1 Mechanisms

Nicotinamide, a poly (ADP-ribose) polymerase inhibitor, is administered prior to STZ injection to partially protect pancreatic *β*-cells from complete STZ-induced destruction. The antioxidant properties of NAD limit β-cell damage to partial injury, resulting in reduced insulin secretion rather than absolute deficiency. This model recapitulates the progressive *β*-cell dysfunction characteristic of non-obese T2D, manifesting as moderate chronic hyperglycemia without significant insulin resistance or obesity (27, 28).

#### 2.3.2 Modeling method

NAD is given before STZ injection to partially protect *β*-cells, leading to reduced insulin secretion rather than absolute deficiency. Typical protocols: rats: STZ 45–65 mg/kg + NAD 85–230 mg/kg; mice: STZ 60 mg/kg + NAD 120 mg/kg, simulating non-obese T2D (27, 29–33).

#### 2.3.3 Advantages and disadvantages

Suitable for studying renal injury related to progressive *β*-cell dysfunction; however, it does not produce significant insulin resistance and obesity, showing a significant difference from the common clinical obese T2D with metabolic syndrome.

#### 2.3.4 Optimization strategies

Consider combining long-term high-fat diet (HFD) on this model basis to introduce insulin resistance factors and construct a more complex pathological background.

### 2.4 Composite injury models

#### 2.4.1 Mechanisms

Uninephrectomy induces hyperfiltration and hyperperfusion in the remaining nephrons, generating hemodynamic stress; high-fat diet induces metabolic disturbances and insulin resistance; low-dose STZ triggers overt hyperglycemia. These three factors act synergistically through the combined effects of hemodynamic injury, metabolic toxicity, and inflammatory pathways to accelerate glomerulosclerosis and interstitial fibrosis (11, 34, 35).

#### 2.4.2 Modeling method

Using combined intervention strategies, mainly including: Streptozotocin (STZ) combined with uninephrectomy (UNx) (STZ + UNx) to accelerate T1D injury; STZ combined with high-fat diet (HFD) (STZ + HFD) to simulate the complete pathology of T2D; and the triple injury model of STZ, high-fat diet combined with uninephrectomy (STZ + HFD + UNx) (23, 25, 36–40).

#### 2.4.3 Advantages and disadvantages

Can significantly shorten the research cycle and accelerate the induction of severe pathological changes; but the mechanisms are confounded (especially UNx introduces non-diabetic nephron reduction and hemodynamic stress), model reproducibility is poor, and surgery increases technical difficulty and animal risks.

#### 2.4.4 Optimization strategies

Explore pharmacological alternatives (such as Ang II sustained release) to replace UNx and reduce confounding factors; apply composite injury protocols to genetic background strains more susceptible to kidney damage; promote the standardization of various intervention factors (sequence, dose, timing).

## 3 Hereditary animal models

### 3.1 Single-gene spontaneous mutation models

#### 3.1.1 Mechanisms

Single-gene spontaneous mutation models develop diabetes through distinct genetic defects that spontaneously manifest at specific ages. Akita mice carry the Ins2C96Y point mutation, causing insulin precursor misfolding, endoplasmic reticulum stress, and *β*-cell apoptosis, leading to early-onset hyperglycemia (41, 42). db/db mice harbor the Leprdb mutation resulting in leptin receptor deficiency and complete loss of hypothalamic leptin signaling, while ob/ob mice carry the Lepob mutation causing absolute leptin deficiency; both exhibit hyperphagia, obesity, insulin resistance, and hyperglycemia. ZSF1 obese rats, generated by crossbreeding, carry both obesity and hypertension susceptibility genes, combining leptin receptor deficiency with a hypertension-prone background to spontaneously develop metabolic syndrome with significant hypertension. Sustained hyperglycemia in all these models subsequently induces renal injury through metabolic disturbances, oxidative stress, and hemodynamic alterations (41, 43–45).

#### 3.1.2 Modeling method

Naturally occurring mutant strains requiring no exogenous induction, with disease phenotypes manifesting spontaneously at specific ages.

#### 3.1.3 Advantages and disadvantages

These models provide stable spontaneous disease platforms without chemical induction artifacts. However, phenotypes often plateau at early to intermediate stages with oversimplified etiology; disease severity is highly strain-dependent; and some models have limited accessibility and high maintenance costs.

#### 3.1.4 Optimization strategies

Apply external stressors such as high-protein diet or uninephrectomy to accelerate late-stage phenotype emergence; optimize genetic backgrounds through strain crossbreeding to obtain more severe phenotypes.

### 3.2 Genetically engineered models

#### 3.2.1 Mechanisms

Genetically engineered models are constructed via molecular biology techniques to precisely manipulate specific genes. OVE26 transgenic mice utilize the rat insulin 2 promoter to drive calmodulin overexpression specifically in pancreatic *β*-cells, causing intracellular calcium dysregulation, β-cell apoptosis, and early-onset severe T1D (46, 47). Endothelial nitric oxide synthase-deficient (eNOS^−^/^−^) mice have reduced nitric oxide production, causing endothelial dysfunction and hypertension; under diabetic conditions, eNOS deficiency further exacerbates glomerular hypertension, oxidative stress, and inflammatory responses, significantly accelerating proteinuria and glomerulosclerosis (48).

#### 3.2.2 Modeling methods

Constructed via modern molecular biology techniques to precisely manipulate gene expression or function. Examples include OVE26 transgenic mice, which overexpress calmodulin in pancreatic *β*-cells, leading to severe early-onset T1D (46, 47), and endothelial nitric oxide synthase-deficient (eNOS) mice, used to study the exacerbating role of endothelial dysfunction in diabetic renal injury when combined with diabetes induction (48, 49).

#### 3.2.3 Advantages and disadvantages

Enable precise mechanistic dissection of specific genes or pathways; some models (e.g., OVE26) recapitulate advanced DKD features. However, they may exhibit non-physiological extreme phenotypes (e.g., extreme hyperglycemia in OVE26) or developmental compensation; construction and maintenance costs are high; and some models have limited strain availability.

#### 3.2.4 Optimization strategies

Apply inducible gene expression systems for temporal control; combine with susceptible genetic backgrounds; use for studying specific mechanistic pathways.

## 4 Traditional Chinese medicine disease-syndrome combination animal models

### 4.1 Mechanisms

These models integrate TCM “syndrome” factors with DKD pathology. Blood stasis syndrome involves microcirculation disorders (50, 51); Qi-Yin deficiency syndrome involves metabolic and immune dysfunction. These factors interact with DKD-associated metabolic, hemodynamic, and inflammatory changes.

### 4.2 Modeling method

#### 4.2.1 DKD blood stasis syndrome model

Based on STZ-induced or db/db spontaneous DKD models, a “blood stasis” state is induced by intravenous injection of high-molecular-weight dextran or housing animals in a low-temperature environment.

#### 4.2.2 DKD Qi-Yin deficiency syndrome model

Following DKD model establishment, intervention is performed via oral administration of qi-consuming and yin-damaging Chinese herbal compounds or a combination of exhaustive swimming and fasting.

### 4.3 Advantages and disadvantages

The main advantage is the integration of modern pathology models with TCM syndrome theory to create “disease-syndrome combination” models for multi-target intervention research. The key disadvantage is the lack of objective syndrome evaluation standards and insufficient specificity in modeling methods.

### 4.4 Optimization strategies

Objective syndrome characterization using multi-omics and imaging technologies. Disease-syndrome homology models using animals with inherent syndrome predispositions (e.g., aged animals). Prescription-syndrome correspondence validation using classic TCM formulas.

### 4.5 TCM monomer research

TCM monomers ameliorate DKD through multi-target pathways including anti-inflammation and anti-oxidation. For example, wogonoside protects podocytes via NF-κB p65-MMP28 axis and renal tubular cells via HNF4A-NRF2 axis. These findings support the multi-target characteristics of TCM and inform disease-syndrome model optimization (52, 53).

## 5 Model mechanisms and selection rationale

DKD models exhibit distinct mechanisms. Among chemically induced models, high-dose STZ directly causes *β*-cell apoptosis via DNA alkylation, suitable for drug screening but with nephrotoxicity. HFD + STZ induces insulin resistance followed by partial *β*-cell damage, mimicking T2D. STZ + NAD uses nicotinamide for DNA repair to partially protect *β*-cells, modeling non-obese T2D.

Among hereditary models, Akita mice carry Ins2 mutation causing insulin misfolding and β-cell toxicity, reducing insulin secretion. db/db and ob/ob mice exhibit leptin pathway defects, presenting obesity, insulin resistance, and hyperglycemia. ZSF1 rats combine leptin deficiency with hypertension, modeling advanced DKD with hypertension. OVE26 mice overexpress calmodulin disrupting insulin secretion, recapitulating advanced T1D-DKD. eNOS-deficient mice have reduced NO synthesis causing endothelial injury, used to study vascular factors in DKD.

Disease-syndrome models integrate blood stasis or Qi-Yin deficiency factors, simulating clinical states for TCM research.

**Figure 1:**
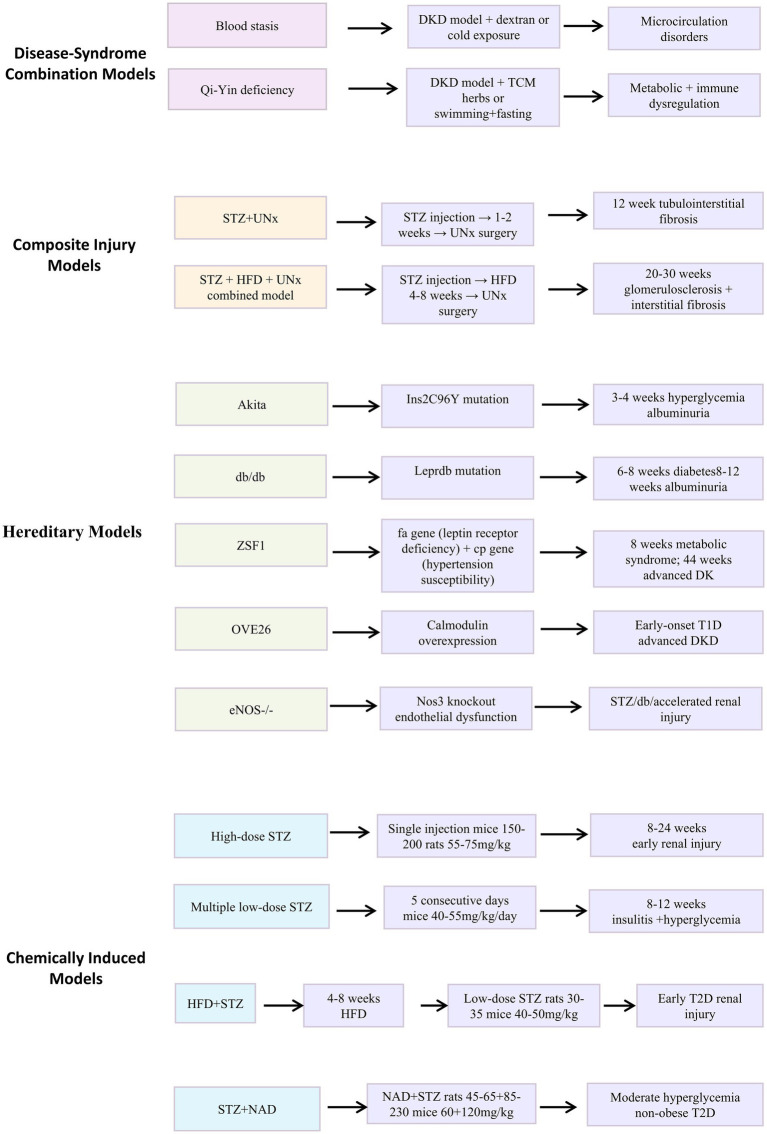
Modeling protocols for major DKD animal models. The figure summarizes the timelines, doses, and intervention sequences for chemically induced, hereditary, composite injury, and disease-syndrome combination models.

*Selection guidance*: HFD + STZ or db/db for early metabolism; ZSF1 or OVE26 for advanced fibrosis; genetically engineered models for cellular mechanisms; disease-syndrome models for TCM evaluation. The modeling protocols for each model are schematically represented in Figure 1, and detailed mechanistic comparisons are provided in Table 1.

**Table 1:** Core mechanisms and applications of major DKD animal models.

Model type	Specific model	Core mechanism	Key pathology	Research application
Chemical	High-dose STZ	β-cell apoptosis via DNA alkylation	Glomerular hypertrophy, microalbuminuria	Drug screening, early T1D
Chemical	HFD + STZ	Insulin resistance + partial β-cell damage	Mesangial expansion, ECM deposition	Early T2D mechanisms, metabolic syndrome
Chemical	STZ + NAD	Nicotinamide promotes DNA repair, partial β-cell protection	Mild renal injury	Non-obese T2D research
Genetic	Akita mice	Ins2 mutation→insulin misfolding, β-cell proteotoxicity	Mesangial expansion, albuminuria	T1D mechanisms
Genetic	db/db mice	Abnormal leptin receptor transcription	Mesangial expansion, albuminuria	Early T2D-DKD
Genetic	ob/ob mice	Leptin deficiency	Mesangial expansion, albuminuria	Obesity-related T2D
Genetic	ZSF1 rats	Leptin deficiency + hypertension background	Glomerulosclerosis, interstitial fibrosis	Advanced DKD with hypertension
Genetic	OVE26 mice	Calmodulin overexpression→insulin secretion disorder	Nodular sclerosis, GFR decline	End-stage T1D-DKD
Genetic	eNOS-deficient	eNOS dysfunction→↓NO synthesis, endothelial injury	Proteinuria, glomerulosclerosis	Vascular mechanisms
TCM	Blood stasis	DKD + microcirculation disorders	Model-dependent	Multi-target TCM research
TCM	Qi-Yin deficiency	DKD + metabolic/immune dysregulation	Model-dependent	TCM formula evaluation

## 6 Discussion

This review systematically examines the main animal models currently used in diabetic kidney disease (DKD) research: chemically and physically induced models, hereditary animal models, and traditional Chinese medicine disease-syndrome combination models. By analyzing the modeling methods, advantages, disadvantages, and optimization strategies for each category, this work reveals the deficiencies in the existing animal model systems and explores potential optimization approaches.

Chemically induced models (e.g., STZ models) are simple to operate and cost-effective, but they suffer from etiological discrepancies and limitations in pathological phenotypes. Hereditary models, while capable of simulating specific genetic defects, often exhibit phenotypic plateaus in single-gene models, and genetically engineered models may produce non-physiological extreme phenotypes. Disease-syndrome combination models attempt to integrate modern medicine with traditional Chinese medicine theory but face challenges in syndrome standardization and model specificity.

The root causes of these model challenges lie in: the mismatch between the simplified design of animal models and the complex pathological processes of human DKD; the disconnection between model evaluation metrics and clinical endpoints; and the differences in disease progression timelines. These limitations directly affect the efficiency of translating preclinical research results into clinical applications.

To bridge this translational gap, a new research paradigm must be established: purpose-driven model selection should be based on specific scientific questions to choose the most suitable model system; proactive optimization strategies need to improve existing models through various means; and clinically aligned evaluation criteria should make animal experimental endpoints more closely reflect clinical reality. Such systematic improvements will help enhance the translational value of DKD research, providing a reliable platform for developing more effective therapeutic strategies.

## 7 Conclusion

In summary, current animal models for diabetic kidney disease still face challenges in simulating disease complexity and predicting clinical outcomes. By establishing a “purpose-oriented” model selection framework, optimizing model development strategies, and refining model evaluation standards, a more comprehensive and predictive research model ecosystem will provide critical support for ultimately overcoming diabetic kidney disease.

## Data Availability

The original contributions presented in the study are included in the article/supplementary material, further inquiries can be directed to the corresponding author.
